# Modern Sociology

**Published:** 1896-12-19

**Authors:** 


### Modern Sociology.

### THE RESURRECTIONIST OF OLD.

It is now more than sixty years since the Anatomy Act was passed, and there are probably few who remember, except as a tradition, the horrors of the preceding time, when the medical schools were supplied with subjects for dissection chiefly by men who stole corpses from the grave. These men were called body-snatchers, or, in a slang phrase, "resurrection men." Respect for the dead made the idea of this violation of the grave horrible to the survivors, and various means were devised to secure that the bodies of the beloved dead should remain undisturbed. The iron coffin, instead of the usual wooden one, was so intended. A heavy iron cage, called a "mortsafe," was another. Mortsafes were of various kinds. Some formed almost a house of iron bars, with a locked gate to it. Others lay flat on the grave, and consisted sometimes entirely of iron, and sometimes of a border of strong masonry with iron bars on the top. But such precautions as these could be taken only by rich men. The poor watched the graves of their dead, night after night, till they might reasonably suppose that putrefaction had set in and the corpse thus become useless for anatomical purposes.

But the body-snatchers were not to be deterred by casual watchers, and the duty of guarding the dead had to be organised as a regular patrol. In villages parties of men took it in turns to keep armed watch, and fired at any suspicious-looking character who was seen in the churchyard after dark. In the village of Symington, in Lanarkshire, there may still be seen a tower from whose vantage the villagers kept a look-out for the resurrection-men, prepared to shoot the first that appeared. In the cemetery of Crail, in Fifeshire, there is a tower, intended (according to Mr. Bailey,* who has prefixed a most interesting monograph on the subject to the curious "Diary of a Resurrectionist") to serve as a receptacle for the bodies until they were too far decayed to be worth stealing.

It must be admitted that medical men were not free from complicity in the crime, and, indeed, medical students were sometimes active agents in it. The excuse was that only the bodies of murderers were permitted to be given to the dissecting-rooms, and the supply of murderers was not sufficient for the needs of the dissecting-room. But the result was that honourable men connived at the rifling of graves. One gentleman, indeed, went further than connivance. The registrar at one of the London schools not only bought bodies from the resurrectionists, but resold them at a higher price to the Edinburgh Medical School. Dismissed from his situation for this practice, he set up a restaurant, but some hint of his former business became known, and spoiled his business as a restaurateur. Dealing with resurrection men was not a very profitable business for the surgeon. Sometimes he had to pay more for the bodies than he could charge his students; but, doubtless, increased lecture fees made up for this. Then at the beginning of the session the resurrection men would wait on him, and promise to supply him with the bodies he needed on condition that they received a certain sum on the spot. Old or sickly people would come to offer him the use of their bodies at death on condition of receiving a pension during their life. If the resurrection men were caught in their work and imprisoned, the surgeon was expected to maintain their families while they were in gaol.

Then penalties were increased. At first convictions were granted only against those who had violated a grave, but latterly the mere fact of having an exhumed body in possession was declared to be a crime, and this threatened every professor of anatomy in the kingdom. The watch over graveyards became more careful, and bodies consequently scarcer and higher in price. One surgeon complained that a corpse that in his student days would have been worth only two guineas fetched latterly sixteen. Then the body-snatchers ceased to confine themselves to buried corpses. The bodies of suicides and others awaiting inquest was stolen, and even from private houses such ghastly thefts were made. Finally, murder was resorted to in order to provide the necessary subjects, and the medical profession had to share in the opprobrium that belonged to the crimes of Burke and Hare. Men of advanced views bequeathed their bodies to the College of Surgeons for the purpose of dissection in the hope of making people show more regard for the necessities of science, but the public horror was not lessened, and there is no doubt that the hands of Government, which would willingly have made reasonable provision for these requirements, were hampered by the superstitious reverence in which the dead were held. But in 1832 the Anatomy Act was passed, and the resurrection man's occupation was gone.

The diary which Mr. Bailey has unearthed deals with the years 1811 and 1812, and there seems to be some doubt as to its authorship, although the editor makes a very plausible surmise that it was one Joseph Naples, who was originally the keeper of a cemetery, but being dismissed from his situation for conniving at body-snatching himself, joined a resurrection gang.

Even when the body was secured the thieves did not always get off safely with their booty. In this connection the present writer is tempted to tell a story current in Ayrshire. Two resurrection men had dug up a body, put it in a sack, and drove away with it in a cart. But on their way home they stopped at a public-house. While they were drinking some wag investigated their baggage, and, finding out its nature, took out the corpse and placed himself in the sack. Presently the thieves came out and drove off. "Man!" exclaimed one present "the corp's moving." "Nonsense," said the other. "But," the first protested, "its warm." And a sepulchral voice answered from the sack, "Aye, ye sinners, and when ye've been as lang in hell as I have, ye'll be warm, tae." Terrified at the thought that they had got a lost soul, the thieves fled, leaving horse and cart to the supposed corpse, who took possession of them. Even if the resurrection men afterwards found out their mistake, it would hardly have been wise to reclaim their property.

* "The Diary of a Resurrectionist, 1811-1812, to which are added an account of the Resurrection Men in London, and a short history of the passing of the Anatomy Act." By James Blake Bailey, B.A., Librarian of the Royal College of Surgeons of England. (London: Swan Sonnenschein and Co.)

